# Ceftriaxone-Loaded Polymeric Microneedles, Dressings, and Microfibers for Wound Treatment

**DOI:** 10.3390/polym15122610

**Published:** 2023-06-08

**Authors:** Pablo Serrano-Castañeda, Miguel Alejandro Ochoa Loyo, Cristian Ezequiel Tinoco Hernández, Brian Miguel Anaya-Ortega, Omar Rodrigo Guadarrama-Escobar, Ericka Anguiano-Almazán, Betsabé Rodríguez-Pérez, Ma. Concepción Peña-Juárez, Alma Vázquez-Durán, Abraham Méndez-Albores, Isabel Marlen Rodríguez-Cruz, Miriam Isabel Morales-Florido, José Juan Escobar-Chávez

**Affiliations:** 1 Unidad de Investigación Multidisciplinaria-Lab 12, Facultad de Estudios Superiores Cuautitlán, Universidad Nacional Autónoma de México, Carretera Cuautitlán-Teoloyucan, km 2.5 San Sebastián Xhala, Cuautitlán Izcalli 54714, Mexico; pabloqfb@hotmail.com (P.S.-C.); ochoaloyomiguel@gmail.com (M.A.O.L.); crashtinoco@gmail.com (C.E.T.H.); miguelanayaortega123@gmail.com (B.M.A.-O.); escobaromarrodrigo@gmail.com (O.R.G.-E.); eri.qa.30@hotmail.com (E.A.-A.); maconcepcionpenajuarez@gmail.com (M.C.P.-J.); mflorido.cf@gmail.com (M.I.M.-F.); 2 Laboratorio de Servicio de Análisis de Propóleos (LASAP), Unidad de Investigación Multidisciplinaria (UIM), Facultad de Estudios Superiores Cuautitlán, Universidad Nacional Autónoma de México, Cuautitlán Izcalli 54714, Mexico; berope380@hotmail.com; 3 Unidad de Investigación Multidisciplinaria L14 (Ciencia y Tecnología de los Materiales), Facultad de Estudios Superiores Cuautitlán, Universidad Nacional Autónoma de México, Cuautitlán Izcalli 54714, Mexico; almavazquez@comunidad.unam.mx (A.V.-D.); albores@unam.mx (A.M.-A.); 4 Unidad de Enseñanza e Investigación, Hospital Regional de Alta Especialidad de Zumpango, Carretera Zumpango-Jilotzingo #400, Barrio de Santiago, 2ª Sección, Zumpango 55600, Mexico; isabelmarlen05@gmail.com

**Keywords:** microneedles, microfibers, dressings, wound treatment, ceftriaxone, Kollicoat^®^ Protect, Kollicoat^®^ MAE 100P

## Abstract

The objective of this study was to create polymeric dressings, microfibers, and microneedles (MN) loaded with ceftriaxone, using PMVA (Poly (Methyl vinyl ether-alt-maleic acid), Kollicoat^®^ 100P, and Kollicoat^®^ Protect as polymers to treat diabetic wounds and accelerate their recovery. These formulations were optimized through a series of experiments and were subsequently subjected to physicochemical tests. The results of the characterization of the dressings, microfibers, and microneedles (PMVA and 100P) were, respectively, a bioadhesion of 281.34, 720, 720, 2487, and 510.5 gf; a post-humectation bioadhesion of 186.34, 831.5, 2380, and 630.5 gf, tear strength of 2200, 1233, 1562, and 385 gf, erythema of 358, 8.4, 227, and 188; transepidermal water loss (TEWL) of 2.6, 4.7, 1.9, and 5.2 g/h·m^2^; hydration of 76.1, 89.9, 73.5, and 83.5%; pH of 4.85, 5.40, 5.85, and 4.85; and drug release (Peppas kinetics release) of n: 0.53, n: 0.62, n: 0.62, and n: 0.66). In vitro studies were performed on Franz-type diffusion cells and indicated flux of 57.1, 145.4, 718.7, and 2.7 µg/cm^2^; permeation coefficient (Kp) of 13.2, 19.56, 42, and 0.00015 cm^2^/h; and time lag (t_L_) of 6.29, 17.61, 27. 49, and 22.3 h, respectively, in wounded skin. There was no passage of ceftriaxone from dressings and microfibers to healthy skin, but that was not the case for PMVA/100P and Kollicoat^®^ 100P microneedles, which exhibited flux of 194 and 0.4 µg/cm^2^, Kp of 11.3 and 0.00002 cm^2^/h, and t_L_ of 5.2 and 9.7 h, respectively. The healing time of the formulations in vivo (tests carried out using diabetic Wistar rats) was under 14 days. In summary, polymeric dressings, microfibers, and microneedles loaded with ceftriaxone were developed. These formulations have the potential to address the challenges associated with chronic wounds, such as diabetic foot, improving the outcomes.

## 1. Introduction

Diabetic foot and wound care affect healthcare systems worldwide. In the United States, wounds affect approximately 8.2 million people, representing an annual cost of $28.1 billion to $96.8 billion per year [1]. The risk of complications from poorly treated wounds, ranging from deep tissue infection to amputation, makes wound care an indispensable health problem to address.

Wounds that have failed to heal in four weeks are defined as “chronic wounds”. Regardless of the origin or cause, these wounds share certain characteristics that make them progress to such a chronic state. This situation not only generates constant pain, discomfort, and mobility limitations but also impacts the social and emotional well-being of the patient [1].

Chronic wounds are in a prolonged and excessive inflammatory stage. These wounds begin with a bacterial infection in the affected area or by hormonal, genetic, nutritional, and venous factors [2].

Treatment of these wounds is complex because of the difficulty of the drug to remain when exudates are present. A substantial part of the drug can also be eliminated, which generates low bioavailability, contrary to what would be expected as the application is topical [3]. In addition, there are other complications, such as the formation of bacterial biofilms, that lower the efficiency of penetration of the active ingredient [4]. Hence, the introduction of microneedles coupled with transdermal systems could be the answer to this problem because they generate microabrasions that allow increasing the flow of drugs. Microneedles are also an answer to ischemic wounds with poor penetration of active ingredients [5], resulting in improved wound healing, promoting tissue remodeling (an important factor for wound healing), and eliminating hazardous sharp waste as dissolvable polymer microneedles are used, thus preventing the potential for injury and transmission of bloodborne infections [6,7].

Microfibers can be incorporated into tissues and fill gaps, such as in the case of diabetic foot, where other formulations have difficult access. Microfibers have mechanical properties, such as elasticity and flexibility, which allow the release of therapeutically interesting active ingredients in a controlled manner, allowing the regeneration of damaged tissue [8].

Similar to fibers, dressings have the advantage of being able to cover larger areas, preventing tissue dehydration, optimizing re-epithelialization, allowing the release of active ingredients at the affected site, providing a temporary protective physical barrier, and absorbing wound drainage [9].

These systems provide advantages, such as localized treatment, safety, easy removal, controlled release of therapeutic agents, avoidance of the first-step effect, improvement of treatment adherence, and the possibility to incorporate growth factors for more efficient recovery of damaged tissue [10,11]. These systems are affordable at the industrial scale. Therefore, generating systems capable of releasing therapeutic agents for wound treatment will allow more efficient wound healing.

The objective of this study was to develop dressings, fibers, and microneedles using PMVA, Kolicoat^®^ 100P, and Kollicoat^®^ Protect. These materials have the potential to enhance the wound-healing process. Although much research has been conducted using other polymers, little information exists on the application of these specific polymers in the creation of dressings, fibers, and microneedles. The intention of developing these formulations is that each one of them can be used for different types of injuries. Therefore, each formulation was evaluated to determine its characteristics. In addition, each formulation was loaded with ceftriaxone, an agent that helps prevent wound infections. In addition, excipients that promote healing were also incorporated. To assess the efficacy of these formulations, comparative in vivo tests were carried out using Recoveron^®^ G. In this way, the ability of the formulations to accelerate the healing process and promote faster and more effective wound recovery can be determined.

## 2. Materials and Methods

The reagents used in the experiment were PMVA (Poly (Methyl vinyl ether-alt-maleic acid), Kollicoat^®^ Protect (BASF, Benito Juárez, Ciudad de México, Mexico), Kollicoat^®^ 100P (BASF, Ciudad de México, Mexico), D-panthenol (BASF, Ciudad de México, Mexico), collagen (Sigma-Aldrich, Toluca, Mexico), propylene glycol (USP), Mili-Q grade distilled water (Millipore Inc., Bedford, MA, USA), N-(2-hydroxyethyl)piperazine-N′-(2-ethane sulfonic acid) (HEPES sodium salt) (Sigma-Aldrich), sodium hydroxide (J. T. Baker, Phillipsburg, NJ, USA), and sodium dibasic phosphate (Fermont, Fremont, OH, USA).

Microneedles and microfibers were manufactured by the micromolding technique of Serrano et al. [12]. The micromolding technique is a simple process, which involves solubilizing the components at 25 °C, then pouring the solution into micromolds, putting the molds in a stainless-steel vacuum chamber with a 3.6 CFM 1/4 HP vacuum pump for 30 min before taking them out, leaving the samples to dry at 25 °C for 72 h, and unmolding them. The technique requires only a few steps and is easy to scale up. The compositions of the formulations are listed in Table 1, and the optimal formulations are shown in Table 9.

Dressings were manufactured by the casting technique of Serrano et al. [13,14]. This technique involves solubilizing the components at 25 °C, then pouring the solution into the molds, leaving the samples to dry at 25 °C for 72 h, and unmolding them.

Characterization tests were previously carried out for the microneedles, microfibers, and dressings to determine the optimal formulations using the following experimental design.

### 2.1. Determination of the Active Content in the Samples

Samples of 5.6 cm^2^ were cut from the dressings and microfibers and dissolved in water to extract the drug. This was performed in extraction tubes, which were constantly agitated at 25 °C for 24 h to ensure the complete extraction of the drug. In the case of the microneedles, their complete arrangement (4 cm^2^) was extracted through the same procedure. Afterward, the samples were filtered and analyzed by UV-visible spectrophotometry at 274 nm.

### 2.2. Bioadhesion Studies

Bioadhesion studies were performed on human skin obtained from abdominoplasties. Circular formulations with an area of 1.27 cm^2^ for microfibers and dressings and 2 cm^2^ for microneedles were used. The samples were placed on the skin, and tests were performed using a texturometer (Brookfield CTB Texture Analyzer, Middleboro, MA, USA) with a load cell of 4500× *g*. The skin with the formulation to be analyzed was placed in the lower part of the texturometer and the test conditions were as follows: a cylindrical probe (perplex cylinder) was applied at a pre-test speed of 2 mm/s until it came into contact with the formulation, a load force of 6.8 gf was applied at a speed of 0.5 mm/s. Finally, the probe was removed at a speed of 4.5 mm/s until a separation distance of 100 mm was obtained, and the force required to remove the formulation from the skin was measured [12,15,16].

### 2.3. Post-Wetting Studies

The procedure was similar to the bioadhesion studies. The difference was that the formulation to be evaluated was moisturized with water, using an atomizer at a distance of 30 cm, 10 min prior to the bioadhesion test [12,15,16].

### 2.4. Resistance of the Dressing and Microfiber to Rupture

The resistance to rupture was evaluated using the texturometer (Brookfield CTB Texture Analyzer) with a load cell of 4500× *g*. Pieces of fibers or dressings with an area of 8.4 cm^2^ were placed on the base of the texturometer and held by tweezers from the top. The test was conducted at pre-test speeds of 2 mm/s and 0.5 mm/s. A tensile force of 6.8 g and a maximum separation distance of 100 mm were applied. The force at which the formulation breaks was determined [12,15].

### 2.5. Microneedle Breaking-Strength Test

The breaking strength was evaluated using the texturometer (Brookfield CTB Texture Analyzer) with a load cell of 4500× *g*. The microneedle array was placed on the circular platform of the texturometer, and a perplex probe was applied downward to measure the force with which the microneedles break [12,15].

### 2.6. Release Studies

Dressing and microfiber samples of 5.6 cm^2^ and microneedles of 2 cm^2^ were used. A USP Apparatus 5 with 500 mL of phosphate buffer (pH = 5.5 to emulate the physiological pH of the skin) [13,16] was used for the tests. The apparatus was set at 37.5 ± 0.2 °C and agitation of 50 rpm. Samples (2 mL) from MN PMVA/100P microfibers, dressing, and microneedles were taken at 3, 6, 9, 12, 16, 20, 25, 30, 35, 40, 45, and 50 min, and 1 and 2 h [12], respectively. For the MN K100P microneedles, sampling was performed at 5, 10, 15, 20, 25, 30, 30, 40, 50, 50, 60, 80, and 100 min, and 2, 2.5, 3, 3.5, 3.5, 4, 4.5, and 5 h, respectively. The amount of drug released was quantified at 274 nm by spectrophotometry to obtain the release profiles and to determine the release kinetics.

### 2.7. In Vitro Percutaneous Absorption Studies

In vitro percutaneous absorption studies were performed using Franz-type diffusion cells with their respective receptor compartment. Human abdominal skin from abdominoplasties was donated by the Hospital San Angel Inn CDMX Mexico and used as a membrane between both compartments. The samples were preserved at −25 ± 0.2 °C for no more than 5 days. The formulations were placed on the skin. The receiving compartment was filled with HEPES buffer solution (pH 7.4) and was kept at 37 °C and 50 rpm. Sampling was performed at 2, 4, 6, 8, 8, 24, 26, 28, 30, and 32 h, respectively, and the drug content was determined spectrophotometrically. The cumulative amount of drug per square centimeter of the patch was plotted as a function of time [13,15].

For the studies with previously damaged skin, the skin was treated by superficial cuts (10 vertical, horizontal, and diagonal cuts) using a scalpel.

### 2.8. In Vivo Studies

Male Wistar rats (healthy rats and diabetic rats) weighing between 200 and 300 g were used, and wounds were generated with a 0.5 mm biopsy punch. The wounds were treated with the proposed formulations and a commercial formulation (Recoveron^®^ G) according to the protocol approved by the ethics committee of the UNAM FESC (code CICUAE-FESC C22_08). The size of the wounds, transepidermal water loss (TEWL), pH (pH meter, Science MED SM-3BW), erythema (Mexameter, C + K Electronic MX 18), and hydration (Corneometer, C + K Electronic CM 825) of the wounds were measured daily during 18 days, using a multiprobe adapter system (C + K Electronic MPA 2, TM 300, Courage + Khazaka electronic GmbH, Köln, Germany).

## 3. Results

The development of the proposed technologies applied different experimental designs. For microneedles, the Box–Behnken and central composite designs were used. For fibers, the central composite design was used. For dressings, the Box–Behnken and central composite designs were applied.

### 3.1. Dressings

**Figure 1:**
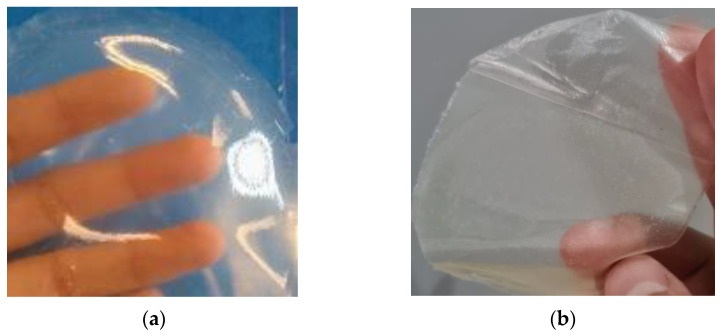
Photographs of the films, where (**a**) is the upper layer with Kollicoat^®^ IR and (**b**) is the lower layer with Kollicoat^®^ Protect.

They are composed of a bottom layer (a central composite design was used) and a top layer (Box–Behnken design). The results are shown in Table 2, Table 3 and Table 7. The factors (x) and response variables (y) for the lower layer were collagen (x1), PEG (x2), erythema (y1), transepidermal water loss (y2), hydration (y3), pH (y4), resistance to rupture (y5), bioadhesion (y6), post-humectation bioadhesion (y7), release at 16 min (y8), and release at 40 min (y9). The dressings are illustrated in Figure 1.

For the upper layer, the factors (x) and response variables (y) were collagen (x1), PEG (x2), D-panthenol (x3), erythema (y1), transepidermal water loss (y2), hydration (y3), pH (y4), rupture strength (y5), bioadhesion (y6), and post-humectation bioadhesion (y7).

### 3.2. Microfibers

**Figure 2:**
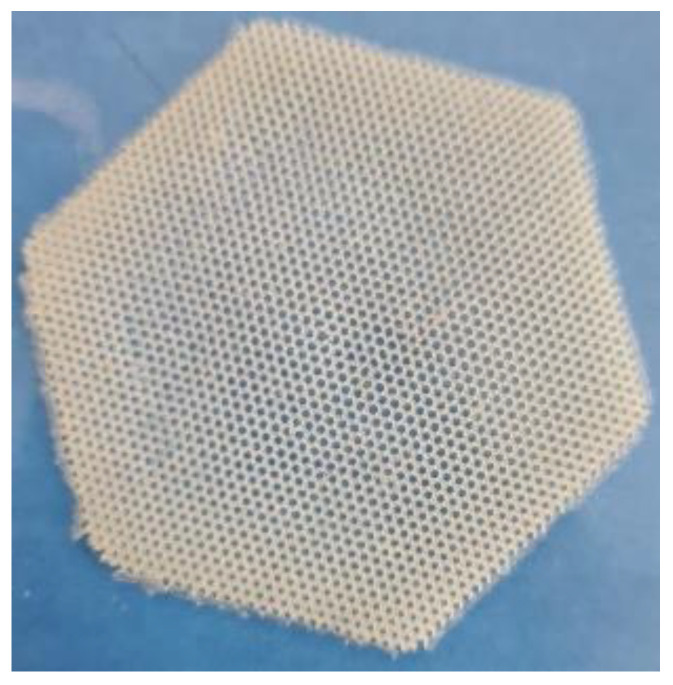
Microfibers with a size of 1 mm; the size was determined using a VE-B1/B0 microscope.

For the fibers, a central composite design was used (Table 4 and Table 7), where the factors (x) and response variables (y) were D-panthenol (x1), PEG (x2), erythema (y1), TEWL (y2), hydration (y3), pH (y4), resistance to rupture (y5), bioadhesion (y6), post-humectation bioadhesion (y7), release at 30 min (y8), and release at 120 min (y9). A photograph of the generated microfibers is shown in Figure 2.

### 3.3. PMVA-Kollicoat^®^ 100P (MN PMVA/100P) and Kollicoat^®^ 100P (MN K100P) Microneedles

**Figure 3:**
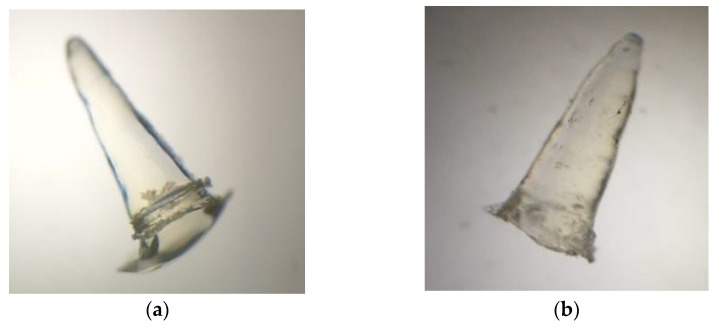
View of the microneedles at 40×: (**a**) MN PMVA/100P and (**b**) MN K100P.

For the microneedles (MN) of PMVA/100P, a Box–Behnken design was used (Table 5 and Table 7) to determine the amount of D-panthenol (x1) and collagen (x2). For both microneedle designs, the dependent variables (responses) were as follows: bioadhesion (y1), post-humectation bioadhesion (y2), rupture strength (y3), pH (y4), erythema (y5), TEWL (y6), hydration (y7), and 50 min release (y8). Photographs of the generated PMVA-Kollicoat^®^ 100P and Kollicoat^®^ 100P microneedles are shown in Figure 3.

A Box–Wilson design was used to optimize the biodegradable polymeric microneedles made of Kollicoat^®^ 100P (MN K100P) (Table 6 and Table 7). The amounts of D-panthenol (x1) and propylene glycol (x2) were chosen as independent variables. The dependent variables (responses) were as follows: bioadhesion (y1), post-dipping bioadhesion (y2), rupture strength (y3), pH (y4), erythema (y5), TEWL (y6), hydration (y7), and 50 min.

### 3.4. Release Studies

Table 7 shows the results of drug release for each formulation (dressing, fibers, and microneedles) evaluated and the optimum formulation.

### 3.5. In Vitro Percutaneous Absorption Studies

Table 8 shows the results of the in vitro percutaneous absorption studies for each formulation applied to damaged skin and healthy skin.

### 3.6. Optimal Formulations

Table 9 shows the optimal formulations for each formulation: dressings, fibers, and microneedles.

### 3.7. In Vivo Studies on Diabetic and Healthy Rats

For the in vivo studies, the dressing formulations, microfibers, and microneedles of PMVEMA-K100P and Kollicoat^®^ 100P, and a commercial pharmaceutical (Recoveron^®^ G) were applied. The skin studies performed on healthy and diabetic rats comprised hydration (Corneometer), erythema (Mexameter), pH (skin pH-meter), and TEWL.

### 3.8. Wound-Healing Time

**Figure 4:**
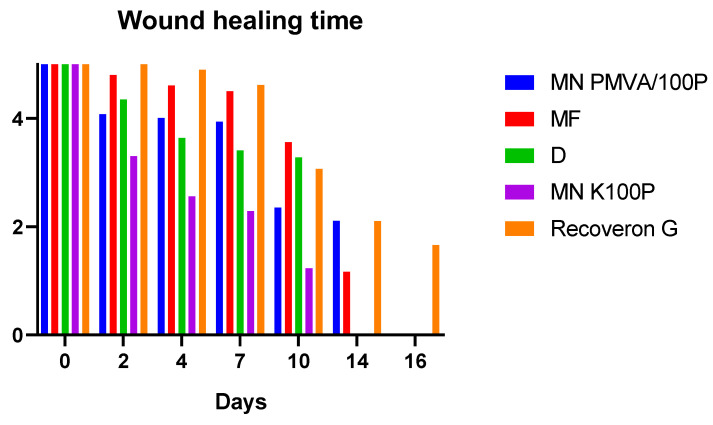
Period (in days) of wound healing in diabetic rats. When the colored line disappears, it indicates that the wound has closed. The nomenclature in the graph is as follows: microneedles of K100P (MN K100P), microfibers (MF), dressing (D), and microneedles of PMVA-K100P (MN PMVA/100P).

**Figure 5:**
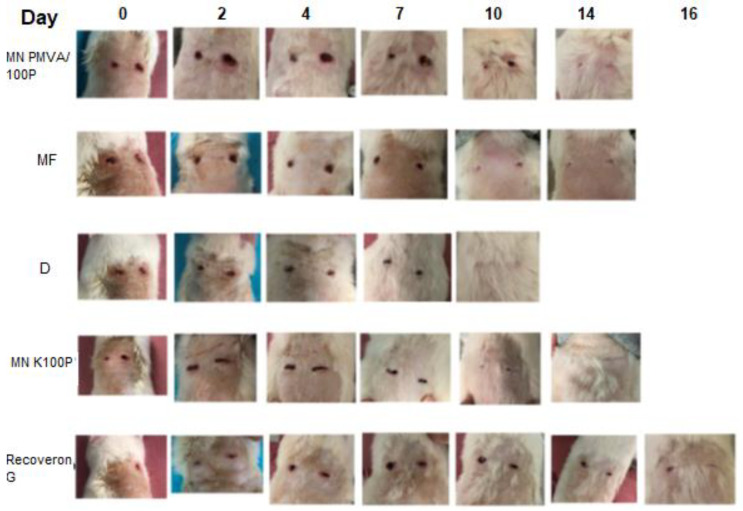
Wound-healing time in diabetic animals for each formulation applied.

**Figure 6:**
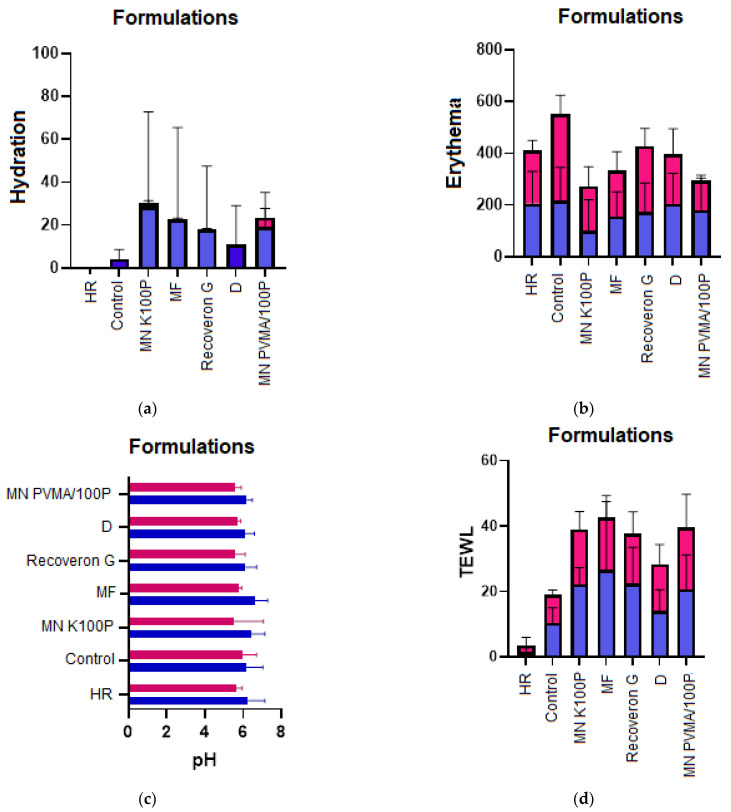
The formulations administered to healthy (blue bars), and diabetic (pink) rats are shown; (**a**) hydration, (**b**) erythema, (**c**) pH, and (**d**) TEWL. The nomenclature in the graph is as follows: healthy rate (HR), wound control (control), microneedles of K100P (MN K100P), microfibers (MF), dressing (D), and microneedles of PMVA-K100P (MN PMVA/100P).

Figure 4 shows the would-healing time. Figure 5 shows the evolution of the wounds in diabetic rats, which, as shown in Figure 6, exhibited more pronounced changes in the parameters (hydration, erythema, pH, and TEWL).

The formulations provided hydration and prevented TEWL. This hydration is due to the components in the formulations (e.g., D-panthenol). In addition, the Kollicoat^®^ (100P and Protect) polymers help avoid the TEWL.

The formulations did not have a significant effect on the pH, which is desirable because pH modifications could make the wound prone to infection and delay the regeneration of the damaged tissue. Likewise, the erythema did not generate irritation; this was the result of components such as D-panthenol and collagen, which aid tissue recovery. A significant reduction (*p*-value < 0.05) in the wound-healing time is observed for the formulations in Figure 5 and Figure 6 compared to the commercial formulation.

## 4. Discussion

### 4.1. Dressings

For the lower layer, collagen was a significant factor as it significantly decreased erythema (*p*-value < 0.05). Himeno et al. [17] showed that collagen can reduce irritation because it decreases inflammation and also has a skin-remodeling function. PEG had a significant quadratic effect that increased the breaking strength because PEG, together with Kollicoat^®^ Protect, reduces the intermolecular forces of the polymer, increasing its mobility and allowing the polymer chains to move more freely, giving it greater flexibility and strength [18]. In terms of bioadhesion, PEG has a moisturizing effect that confers adhesive properties because the formation of hydrogen bridges allows better interaction between the formulations and the substrate, which in this case, is the skin [19].

The factors that were significant in the top layer (α < 0.05) were D-panthenol, which decreases the breaking strength because it interacts with the polymeric chain, making it more brittle, and the interaction between PEG and D-panthenol, which increases skin hydration because both are moisturizing agents [19].

The release kinetics of formulations 1, 2, 3, 6, 10, 13, 16, 17, and 18, respectively, and the optimal formulation followed an anomalous transport mechanism (0.50 > n < 1.0), indicating that there is a diffusion and swelling process that controls the release. Formulations 4, 5, 7, 8, 8, 9, 11, 13, 14, 15, and 19, respectively, showed n > 1, and exhibited a super case II release mechanism, which is characteristic of the diffusion and relaxation of the polymeric chains, with the subsequent erosion of the polymer, which allows the release of the active compound from the matrix [12,20]. For the in vitro studies on Franz-type cells, the optimal formulation yielded the following parameters: in wounded skin, a flow of 57.7 µg/cm^2^·h, Kp of 13.2 cm^2^/h, and T_L_ of 6.29 h, whereas in healthy skin, there was no passage of the drug, which allowed the administration of the minimum inhibitory concentration (50 µg) to the wounds [21].

Regarding bioadhesion and post-humectation bioadhesion, the optimized dressing presented bioadhesive characteristics despite being wet as a result of its components, such as PEG and D-panthenol [19], which moisturize the skin and allow the formulation to adhere to the substrate through hydrogen bonds. In addition, the dressing does not allow TEWL, thanks to the polymer used. D-panthenol provides hydration to the tissue, reducing erythema by reducing inflammation [22] in the area and having a slightly acidic pH that is compatible with the skin. D-panthenol is also the precursor of pantothenic acid [23].

### 4.2. Microfibers

The significant factors (*p*-value < 0.05) for microfibers are described below. D-panthenol decreased irritation but increased water loss because this compound is a precursor of vitamin B5, which confers hydration properties and reduces erythema by reducing inflammation [22]. PEG had a quadratic effect on the decrease in post-wetting because of the increase in plasticizer in the polymer. As a result, bioadhesive action was reduced [24]. The optimal formulation followed Peppas release kinetics. Formulations 1 and 3–7 exhibited an abnormal transport mechanism with an n value between 0.50 and 1.0, indicating that diffusion and swelling control release. For the remaining formulations (8–10, n > 0.89), the super case II mechanism applies, indicating that there is a process of diffusion and relaxation of the polymer chains with polymer erosion that allows the release of the active ingredient from the matrix [12,20].

In the in vitro studies on Franz-type cells, the optimal fiber formulation applied to damaged skin yielded the following parameters: a flow of 145.4 µg/cm^2^·h, Kp of 19.5693 cm^2^/h, and T_L_: 17.61 h. No drug passed through healthy skin. Therefore, the minimum inhibitory concentration (50 µg) can be administered to the wounds [21]. Regarding bioadhesion and post-humectation bioadhesion, a bioadhesive system was generated, which showed minimum TEWL, although this depends on how many layers of microfibers are applied onto the skin, hydrating the tissue, preventing erythema, and having a slightly acidic pH that is compatible with the skin.

### 4.3. Microneedles

The D-panthenol factor was significant (*p*-value < 0.05) and showed a quadratic effect that decreased the resistance to rupture. D-panthenol acts as a plasticizer, reducing the resistance, although its main function is hydration. In the case of PMVA with PEG, the latter acts as a plasticizer, lowering the resistance to rupture. The influence of water causes the glass transition temperature (Tg) of the polymer to change as a function of humidity because the solvent is compatible with the polymer, and there is a decrease in the Tg, causing it to reach its elastomeric state [25]. In an extreme case, hydrolysis can occur, causing a rupture of the molecular chains, loss of molecular weight, and, therefore, loss of the properties of the polymer. This is an irreversible process [26]. In the TEWL response, D-panthenol significantly (*p*-value < 0.05) increased the TEWL because D-panthenol is a moisturizing agent and absorbs water from the environment, thus providing the necessary moisturization to the skin [22]. D-panthenol also significantly increased (*p*-value < 0.05) the hydration response. A good humectant must have a sufficient degree of hygroscopicity to absorb moisture from the atmosphere and retain it in the face of possible fluctuations in humidity. Collagen significantly (*p*-value < 0.05) decreased drug release. Studies have determined that collagen can generate sustained release systems, which delay the release of the active ingredients [27]. Similarly, transdermal collagen patches have been developed to control drug release [28]. This effect is due to the cross-linking of collagen with the polymer, which can generate multiple hydrogen bonds. These bonds are much stronger than van der Waals bonds, although they are weaker than covalent bonds because there are many of them between the polymer chains, resulting in greater attractive forces between the polymers, which hinder the release of active ingredients [29]. The microneedles followed Peppas release kinetics, indicating an anomalous transport mechanism 0.50 > n < 1.0 involving diffusion and swelling processes that control the release [12,20]. In the in vitro studies on Franz-type cells, the optimal formulation yielded the following parameters: a flow of 1.6 µg/cm^2^·h, Kp of 0.00009 cm^2^/h, and T_L_: 20.8 h for wounded skin and a flow of 1.2 µg/cm^2^·h, Kp of 0.00007 cm^2^/h, and T_L_ of 28 h for healthy skin. The microneedles with an area of 2.6 cm^2^ allowed the administration of the minimum inhibitory concentration (50 µg) to the wounds [21].

The factors that affected the microneedles (Kollicoat^®^ 100P) were D-panthenol (hydration) and PEG (an increase in the resistance to rupture) by interacting with the polymeric chain, making it more flexible. Kollicoat^®^ 100P is a copolymer of methacrylic acid with ethyl acrylate that forms an inert matrix and, therefore, generates a solid porous network where diffusion processes occur. According to the Peppas mechanism (n between 0.45 and 0.8939), release from this formulation exhibits a non-Fickian process, indicating that there is diffusion and swelling that control the release [12,20]. In the in vitro studies on Franz-type cells, the optimal formulation yielded the following parameters: a flow of 4 µg/cm^2^·h, Kp of 0.00002 cm^2^/h, and T_L_ of 22.3 h on wounded skin and a flow of 2.7 µg/cm^2^·h, Kp of 0.00001 cm^2^/h, and T_L_ of 9.8 h on healthy skin. Thus, the microneedles with an area of 2.6 cm^2^ allowed the administration of the minimum inhibitory concentration (50 µg) to the wounds [21].

### 4.4. In Vivo Studies on Diabetic and Healthy Rats

The hydration test is performed to demonstrate the moisturizing efficacy of the active ingredients. The integral treatment of a patient with chronic wounds, whatever the etiology of these, must address certain general principles. The application of moisturizing substances is highly recommended, both for wound care and for the prevention of new lesions, and results in an improvement in healing and a decrease in pain [30]. Therefore, the results obtained are considered promising for wound care. Figure 6 illustrates how the formulations generate hydration in the wounds for both diabetic and healthy rats compared to the control wound and healthy skin. This outcome is due to the D-panthenol and PEG that the formulations contain, which provide hydration to the tissue [18,19].

### 4.5. Hydration

The hydration test results revealed a state of dehydration, which results in skin that appears dull, rough, and lacking in suppleness. The integral treatment of a patient with chronic wounds, whatever the etiology of these, must address certain general principles. The application of moisturizing substances is highly recommended, both for wound care and for the prevention of new lesions, and results in an improvement in healing and a decrease in pain [30]. Therefore, the results obtained are considered promising for wound care. Figure 6a illustrates how the formulations generate hydration in the wounds for both diabetic rats and healthy rats compared to the control wound and healthy skin.

### 4.6. Erythema

Redness and swelling are symptoms of various infectious and skin diseases. In dermatology, the erythema test is used for objective clinical diagnoses, allergy tests, melanoma measurements, and determining the scar color [30]. Figure 6b illustrates how the formulations decrease irritation in comparison with healthy skin and the control wound. The microneedle formulations were the best performing. Application of these formulations decreases erythema compared with the control wound. This is due to the components of the formulations, such as D-panthenol and collagen, which have been shown to reduce inflammation and improve the healing process [17,22].

### 4.7. pH

Biofilm growth in a wound generates a change in the pH of the epidermis to either acidic or alkaline, but most commonly from acidic to alkaline [30,31], which is an early indicator of infection. Therefore, reducing the pH levels (i.e., having an acidic pH) would improve the condition of the wound [30]. Clinical studies carried out in diabetic patients with wounds demonstrated a reduction in the bacterial load with the application of acidic ointments [31]. Thus, management of the pH by the microneedle array would not negatively affect situations where the dermis is exposed and would promote tissue regeneration, and reduce complications that may occur in a chronic wound. Figure 6c shows that the formulations maintain a slightly acidic character in the wounds, although their effect is not significant (*p*-value > 0.05).

### 4.8. Transepidermal Water Loss (TEWL)

TEWL is an indispensable parameter for analyzing the skin’s barrier function. The basis of this process is the diffusion of water in the stratum corneum. The measurement of TEWL makes it possible to evaluate the intrinsic barrier function of the stratum corneum. A high TEWL value indicates dysfunction or deterioration of the barrier function [30,32]. Figure 6d shows how the formulations that contain a wetting agent, such as D-panthenol absorb water from the environment, so the TEWL test detects a greater water loss in tissues that have more water. Nevertheless, the formulations using Kollicoat^®^ prevent water loss.

## 5. Conclusions

The polymers used in the project (PMVA, Kollicoat^®^ 100P, and Kollicoat^®^ Protect) can be used to manufacture dressings, microfibers, and microneedles with adequate physicochemical and biopharmaceutical characteristics for application to the skin. The optimal formulations are listed in Table 9. Table 1 lists the components of each formulation. The technologies developed proved to have better in vivo tissue regeneration properties than the commercial pharmaceutical Recoveron^®^ G. This suggests that the tissue regeneration capacity of the active ingredients used in the proposed formulations may be greater than that of the commercial one. However, other active ingredients of therapeutic interest could also be applied.

## Figures and Tables

**Table 1:** Proposed formulations.

Low Layer Inferior	Mount	Top Layer Dressing	Mount	Fibers	Mount	Microneedles PMVA Kollicoat 100P^®^	Mount	Microneedles Kollicoat 100P^®^	Mount
Kollicoat Protect^®^	1000 mg	Kollicoat IR^®^	1000 mg	Kollicoat Protect^®^	1000 mg	PMVA	300 mg	Kollicoat 100P^®^	400 mg
Collagen	117.16–682.84 mg	Collagen	200–600 mg	Collagen	500 mg	Kollicoat 100P^®^	400 mg	Collagen	250 mg
PEG	117.16–682.84 µL	PEG	117.16–682.84 µL	PEG	58.58–341.42 µL	Collagen	250–500 mg	D-panthenol	29.29–170.71 mg
Drug	500 mg	D-panthenol	10%	Drug	500 mg	PEG	150 µL	PEG	23.43–136.57 µL
Hyaluronic Acid	1%	Water	10 mL	D-panthenol	2.93–17.07%	D-panthenol	2–6%	Drug	500 mg
D-panthenol	4%			Water	5 mL	Drug	500 mg	Water	5 mL
Water	10 mL					Water	5 mL		

**Table 2:** Central composite design bottom layer of dressings.

Formulation	X1	X2	Y1	Y2	Y3	Y4	Y5	Y6	Y7
	mg	µL		g/h·m^2^	%		g.f	g.f	g.f
1	400	400	89	0	48.1	5.51	1200	261	257
2	200	600	119	0.2	47.1	5.65	1002	261	296.5
3	400	682.843	104	3	60.1	5.9	1619	274	312
4	400	400	118	1.3	40.1	5.45	1002	261	313.5
5	682.843	400	98	0	51.9	5.39	1700	280	209
6	400	117.157	103	4.8	47.9	5.86	2436	274	312
7	200	200	117	0.3	48.3	5.51	1719	261	301.5
8	400	400	82	0.4	52.1	5.74	1011.5	230	298
9	400	400	53	0.7	54.9	5.39	1432	261	368.5
10	400	400	56	5.7	60.9	6.04	1335	246.5	313.5
11	682.843	400	127	0	46.9	5.55	1719	261	280
12	600	600	80	3.3	47.3	5.96	1244	274	312
13	400	400	80	0	54.6	5.92	1483	209	313.5
14	400	400	132	2.5	65.8	6.02	1076	246.5	230
15	600	200	102	4.7	48	5.42	2414.5	246.5	313.5
16	117.157	400	130	3.2	49	5.83	1533	230	298
17	117.157	400	137	4.4	58.1	5.89	1898.5	230	305.5
18	400	400	115	0	33.6	5.7	1623	221.5	298
19	400	400	81	0.5	52.7	5.52	1623	261	298
Optimal low layer	0.4995	125	32	4.15	67.5	5.48	1320	289	299
Optimal both layers	0.4995	125	358	2.6	76.1	5.72	2200	281.34	186.34

**Table 3:** Box–Behnken design top layer of the dressing.

Formulation	X1	X2	X3	Y1	Y2	Y3	Y4	Y5	Y6	Y7
	mg	µL	%		g/h·m^2^	%		g.f	g.f	g.f
1	400	300	30	231	0	64.8	5.1	194	340	370
2	400	100	10	339	0	60.8	5.01	868	206	267
3	200	200	20	762	0	51.2	4.23	1150	193.5	235
4	600	200	30	248	0	61.4	3.58	660	258	355
5	400	200	20	273	0	41.5	5.21	1180	214.5	170
6	200	200	10	820	0.1	45.7	5.51	1348	219.5	200
7	400	200	20	766	1	37.8	6.09	825	240	295
8	600	200	10	263	1.6	44.7	6.4	1480	240	294
9	600	300	20	850	1.2	32.2	6.33	700	239	277.5
10	400	300	10	399	3.6	43.6	6.62	1280	345	295
11	600	100	20	288	4.3	43.7	6.8	1190	239	205
12	400	100	30	657	1.2	39.6	6.91	295	315	330
13	200	300	20	350	0	56.3	6.59	625	365	245
14	200	200	30	275	0	39.4	6.41	570	268	227.5
15	200	100	20	230	3.9	37.1	6.79	1820	285	200
16	400	200	20	259	0	53.5	6.94	510	370	300
17	400	200	20	254	0	51	6.79	390	390	260
Optimal top layer	490	125	10	338	3.04	66.3	5.6	1104	227	265

**Table 4:** Central composite fiber design.

Formulation	X1	X2	Y1	Y2	Y3	Y4	Y5	Y6	Y7
	%	µL		g/h·m^2^	%		g.f	g.f	g.f
1	10	200	101	8.4	96.3	5.35	1661.5	430	700
2	15	300	42	13.3	89.8	5.73	1706.5	428	550
3	17.07	200	0	12.6	70.1	5.36	110	430	440
4	10	341.42	85	8.9	79.3	5.54	1750	390	500
5	10	58.58	81	7.3	70.8	5.42	1400	510	450
6	5	300	104	0	58.8	5.47	800	520	610
7	10	200	87	0	72.7	5.51	850	620	620
8	5	100	112	0.6	55.2	5.53	2700	500	430
9	2.93	200	168	0.6	68.7	5.53	1390	420	560
10	15	100	104	7	82.9	5.53	2400	390	500
Optimal	9.3	192	8.4	4.7	89.9	5.4	1233	720	831.5

**Table 5:** Box–Behnken design of MN PMNVA/100P.

Formulation	x1	x2	y1	y2	y3	y4	y5	y6	y7
	%	mg	g.f	g.f	g.f			g/h·m^2^	%
1	6	375	479	939.5	0	4.86	439	0.6	80.7
2	4	375	655	760	4491	4.98	214	0.4	46.5
3	4	500	569	677.5	4494	4.88	209	0	46.5
4	4	375	657	702.5	4459	4.72	241	0	36.2
5	6	500	684	701	0	5.31	256	0.5	60.5
6	6	250	584	918.5	0	5.2	251	1	60.7
7	2	375	611	913	4551	5.84	226	0.2	52.6
8	2	500	565	1130	4549	5.02	279	0.4	39.5
9	4	250	549	1038	4528	5.15	216	0	35.4
10	4	375	812	1347	4450	5.39	216	0	39.8
11	4	375	572	1204	4460	5.45	258	0	50.9
12	2	250	954	1081	4567	6.14	250	0.6	50.2
Optimal	2	288	2487	2380	1562	5.85	227	1.9	73.5

**Table 6:** Central composite design of MN K100P.

Formulation	x1	x2	y1	y2	y3	y4	y5	y6	y7
	mg	µL	g.f	g.f	g.f			g/h·m^2^	%
1	100	136.569	862	450	316.5	5.29	108.666	3.46	89.53
2	100	80	833.5	453	393.5	4.64	33	3.83	99.23
3	50	120	876.5	500	329	5.52	41.33	3.8	89.93
4	170.711	80	854	750	393	4.41	53	0.56	76.52
5	100	23.4315	539	500	404	6.31	20.66	1.26	33.96
6	29.2893	80	557	907	466.5	5.99	35	1.6	33.46
7	150	40	572	1100	372.5	5.11	35.66	0.4	34
8	100	80	572	860	341	4.67	104	1.36	39.03
9	50	40	572	868	440	7.4	25	6.26	76.43
10	150	120	518	560	311.5	5.83	8	3.26	99.36
11	100	80	420	570	431	5.77	0	3.6	66.5
Optimal	64.04	73.7304	510.5	630.5	385	4.85	188	5.2	83.5

**Table 7:** Results of drug release evaluation for each formulation.

Dressing Formulation	Y8	Y9	Fiber Formulation	Y8	Y9	Formulation MN PMVA/100P	y8	Formulation MN K100P	y8
	%	%		%	%		%		%
1	56.87	72.6	1	77.5	88.67	1	27.91	1	10
2	66.47	75.74	2	77.84	90.15	2	24.45	2	8.9
3	67.2	78.01	3	106.66	101.09	3	30.12	3	7.8
4	70.05	87.87	4	91.01	101.77	4	21.32	4	9.2
5	70.3	82.72	5	92.29	100.08	5	25.88	5	8
6	70.35	99.12	6	82.18	97.74	6	34.09	6	9.4
7	70.49	88.49	7	74.33	89.85	7	32.74	7	8.6
8	72.51	86.69	8	86.34	88.32	8	32.44	8	8.2
9	76.56	91.61	9	78.49	88.66	9	54.5	9	7.9
10	78.73	97.16	10	84.81	99.67	10	29.43	10	4.5
11	82.82	84.39	Optimal	75.58	88.88	11	31.18	11	5.5
12	83.91	99.82				12	41.37	Optimal	25.9
13	85.22	99.5				Optimal	25.91		
14	85.67	99.51							
15	87.49	99.46							
16	90.8	99.34							
17	91.11	97.15							
18	92.11	98.3							
19	92.57	92.43							
Optimal low layer	68.5	84.25							
Optimal both layers	71.06	92.4							

**Table 8:** Studies In vitro percutaneous absorption studies.

	Skin	Flow	Kp	TL
		µg/cm^2^·h	cm^2^/h	h
Dressings	Damaged skin	57.7	13.2	6.29
	Healthy skin	No drug passed through the skin		
fibers	Damaged skin	145.4	19.57	17.61
	Healthy skin	No drug passed through the skin		
PMVEMA	Damaged skin	1.6	9 × 10^−5^	20.8
	Healthy skin	1.2	7 × 10^−5^	28
Kollicoat 100P	Damaged skin	4	2 × 10^−5^	22.3
	Healthy skin	2.7	1 × 10^−5^	9.8

**Table 9:** Optimal formulations.

Low Layer Inferior	Mount	Top Layer Dressing	Mount	Fibers	Mount	Microneedles PMVA	Mount	Microneedles	Mount
						Kollicoat 100P^®^		Kollicoat 100 P^®^	
Kollicoat Protect^®^	1000 mg	Kollicoat IR^®^	1000 mg	Kollicoat Protect^®^	1000 mg	PMVA	300 mg	Kollicoat 100P^®^	400 mg
Collagen	117.157 mg	Collagen	490 mg	Collagen	500 mg	Kollicoat 100P^®^	400 mg	Collagen	250 mg
PEG	400 µL	PEG	125 µL	PEG	192 µL	Collagen	288 mg	D-panthenol	64.04 mg
Drug	500 mg	D-panthenol	10%	Drug	500 mg	PEG	150 µL	PEG	73.73 µL
Hyaluronic Acid	1%	Water	10 mL	D-panthenol	9.30%	D-panthenol	2%	Drug	500 mg
D-panthenol	4%			Water	5 mL	Drug	500 mg	Water	5 mL

## Data Availability

No new data were created or analyzed in this study. Data sharing is not applicable to this article.

## References

[1] Sen C.K. (2019). Human Wounds and Its Burden: An Updated Compendium of Estimates. Adv. Wound Care.

[2] Beyene R.T., Derryberry S.L., Barbul A. (2020). The Effect of Comorbidities on Wound Healing. Surg. Clin..

[3] Derakhshandeh H., Aghabaglou F., McCarthy A., Mostafavi A., Wiseman C., Bonick Z., Ghanavati I., Harris S., Kreikemeier-Bower C., Moosavi Basri S.M. (2020). A Wirelessly Controlled Smart Bandage with 3D-Printed Miniaturized Needle Arrays. Adv. Funct. Mater..

[4] Xu J., Danehy R., Cai H., Ao Z., Pu M., Nusawardhana A., Rowe-Magnus D., Guo F. (2019). Microneedle Patch-Mediated Treatment of Bacterial Biofilms. ACS Appl. Mater. Interfaces.

[5] Yao S., Luo Y., Wang Y. (2022). Engineered microneedles arrays for wound healing. Eng. Regen..

[6] Sabbagh F., Kim B.-S. (2023). Ex Vivo Transdermal Delivery of Nicotinamide Mononucleotide Using Polyvinyl Alcohol Microneedles. Polymers.

[7] Yu X., Wang C., Wang Y., Li L., Gao X., Zhu T., An P., Meng Z., Wang W., Wu T. (2022). Microneedle Array Patch Made of Kangfuxin/Chitosan/Fucoidan Complex Enables Full-Thickness Wound Healing. Front. Chem..

[8] Lanno G.M., Ramos C., Preem L., Putrinš M., Laidmäe I., Tenson T., Kogermann K. (2020). Antibacterial Porous Electrospun Fibers as Skin Scaffolds for Wound Healing Applications. ACS Omega.

[9] Obagi Z., Damiani G., Grada A., Falanga V. (2019). Principles of Wound Dressings: A Review. Surg. Technol. Int..

[10] Ray P., Singh S., Gupta S. (2019). Topical Antimicrobial Therapy: Current Status and Challenges. Indian J. Med. Microbiol..

[11] Serrano-Castañeda P., Escobar-Chavez J.J., Rodriguez-Cruz I.M., Melgoza L.M., Martinez-Hernandez J. (2018). Microneedles as Enhancer of Drug Absorption Through the Skin and Applications in Medicine and Cosmetology. J. Pharm. Pharm. Sci. A Publ. Can. Soc. Pharm. Sci. Soc. Can. Des Sci. Pharm..

[12] Castañeda P.S., Domínguez Delgado C.L., Cruz I.M.R., Contreras L.M.M., Trinidad E.M.M., Cervantes M.L., Escobar-Chávez J.J. (2020). Development of Poly (Methyl vinyl ether-alt-maleic acid) Microneedles for Transdermal Delivery of Atorvastatin Calcium. Curr. Pharm. Biotechnol..

[13] Castañeda P.S., Escobar-Chávez J.J., Aguado A., Cruz I., Contreras L.M.M. (2017). In Design and evaluation of a transdermal patch with atorvastatin. Farmacia.

[14] Castañeda P.S., Escobar-Chávez J.J., Vázquez J.A., Cruz I.M.R., Contreras L.M.M. (2020). Pravastatin Transdermal Patches: Effect of The Formulation and Two Different Lengths of Microneedles on In-vitro Percutaneous Absorption Studies. Iran J. Pharm. Res..

[15] Almazan E.A., Castañeda P.S., Torres R.D., Escobar-Chavez J.J. (2020). Design and Evaluation of Losartan Transdermal Patch by Using Solid Microneedles as A Physical Permeation Enhancer. Iran J. Pharm. Res..

[16] Escalona-Rayo C.F., Serrano-Castañeda P., López-Cervantes M., Escobar-Chávez J.J. (2019). Optimization of Unidirectional Mucoadhesive Buccal Patches Based on Chitosan and Pluronic® F-127 for Metoprolol Controlled Release: In Vitro and Ex Vivo Evaluations. J. Pharm. Innov..

[17] Himeno A., Tsujikami M., Koizumi S., Watanabe T., Igase M. (2022). Effect of Reducing Pigmentation by Collagen Peptide Intake: A Randomized, Double-Blind, Placebo-Controlled Study. Dermatol. Ther..

[18] Korigodskii A.A., Zhirnov A.E., Kechekyan A.S., Zezin S.B. (2022). Transparent Polymer Blends of Poly(methyl methacrylate) and Poly(propylene glycol). Polymers.

[19] Panda N., Jena S., Kumar P.R., Pradhan M.A., Satpathy P.R., Mishra MM C. (2023). Evaluation and Characterization Of Bioadhesive Drug Delivery Systems. J. Pharm. Negat. Results.

[20] Pourtalebi Jahromi L., Ghazali M., Ashrafi H., Azadi A. (2020). A comparison of models for the analysis of the kinetics of drug release from PLGA-based nanoparticles. Heliyon.

[21] Poojary A., Kumar Bari A., Kokare R., Pereira J., Rohra S. (2020). Minimum inhibitory concentration (MIC) of ceftriaxone for blood culture isolates of nalidixic acid resistant Salmonella (NARS)—A 9 year study. Int. J. Infect. Dis..

[22] Proksch E., de Bony R., Trapp S., Boudon S. (2017). Topical use of dexpanthenol: A 70th anniversary article. J. Dermatol. Treat..

[23] Shin J.Y., Kim J., Choi Y.H., Kang N.G., Lee S. (2021). Dexpanthenol Promotes Cell Growth by Preventing Cell Senescence and Apoptosis in Cultured Human Hair Follicle Cells. Curr. Issues Mol. Biol..

[24] Chen S., Zhang K., Li Z., Wu Y., Zhu B., Zhu J. (2023). Hydrogen-bonded supramolecular adhesives: Synthesis, responsiveness, and application. Supramol. Mater..

[25] Wang S., Liu R., Fu Y., Kao W.J. (2020). Release mechanisms and applications of drug delivery systems for extended-release. Expert Opin. Drug Deliv..

[26] Tarvainen M., Sutinen R., Somppi M., Paronen P., Poso A. (2001). Predicting plasticization efficiency from three-dimensional molecular structure of a polymer plasticizer. Pharm. Res..

[27] Peers S., Montembault A., Ladavière C. (2020). Chitosan hydrogels for sustained drug delivery. J. Control. Release.

[28] Terzopoulou Z., Michopoulou A., Palamidi A., Koliakou E., Bikiaris D. (2020). Preparation and Evaluation of Collagen-Based Patches as Curcumin Carriers. Polymers.

[29] León-López A., Morales-Peñaloza A., Martínez-Juárez V.M., Vargas-Torres A., Zeugolis D.I., Aguirre-Álvarez G. (2019). Hydrolyzed Collagen-Sources and Applications. Molecules.

[30] Mohd Ariffin N.H., Hasham R. (2020). Assessment of non-invasive techniques and herbal-based products on dermatological physiology and intercellular lipid properties. Heliyon.

[31] Sim P., Strudwick X.L., Song Y., Cowin A.J., Garg S. (2022). Influence of Acidic pH on Wound Healing In Vivo: A Novel Perspective for Wound Treatment. Int. J. Mol. Sci..

[32] Klotz T., Ibrahim A., Maddern G., Caplash Y., Wagstaff M. (2022). Devices measuring transepidermal water loss: A systematic review of measurement properties. Ski. Res. Technol..

